# Analyzing Forged Quality of Thin-Walled A-286 Superalloy Tube under Multi-Stage Cold Forging Processes

**DOI:** 10.3390/ma16134598

**Published:** 2023-06-26

**Authors:** Liang Tao, Zhiguo Feng, Yulian Jiang, Jinfang Tong

**Affiliations:** 1 School of Mechanical Engineering, Guizhou University, Guiyang 550025, China; happyskytl@163.com (L.T.); yljiang1219@163.com (Y.J.); jftong2020@163.com (J.T.); 2 School of Mechanical Engineering, Guizhou Institute of Technology, Guiyang 550003, China; 3 Key Laboratory of Special Equipment and Manufacturing Technology, Guizhou University, Guiyang 550025, China

**Keywords:** cold forging, thin-walled tube, A-286 superalloy, forging quality

## Abstract

Cold forging is suitable for manufacturing thin-walled tubes; however, a poorly planned forging process results in serious quality problems. This paper aims to determine an appropriate cold forging process for thin-walled A286 superalloy tube with ideal forming quality. We analyzed the effects of the two forging processes with reverse forging sequences on forming defects and hardness distribution in the thin-walled tubes via finite element simulation. The methods of optical microscope, micro-hardness, scanning electron microscope, and electron-backscattered diffraction were used to validate the tube forming quality. The simulation results revealed that the Type-I process was an appropriate forging process for meeting the quality requirements. For the Type-I process, an underfilling defect was observed at the bottom of the rod section of the tube. The stress concentration in the head section was lower than that in the Type-II process, potentially reducing the probability of crack initiation. Compared to the rod section, the head section may exhibit higher hardness magnitudes due to the greater strain distribution. The experimental results confirmed the feasibility of the Type-I process. The increased hardness in the head section may be primarily attributed to the more intense plastic deformation applied to the material in this section by the Type-I process.

## 1. Introduction

Thin-walled tubes are extensively used in aviation, aerospace, and automobile industries since they are lightweight [1,2,3]; however, they are difficult to machine due to their weak mechanical properties. Generally, both warm and cold forging technologies are suitable for manufacturing thin-walled tubes, circumventing the aforementioned limitations. On the one hand, warm forging reduces both the forging load and forging time, but oxidation occurs on forged part surfaces [4,5,6]. On the other hand, parts formed by cold forging have lower costs, good surface quality, improved mechanical properties, and high dimensional accuracy [7,8,9]. Therefore, cold forging is more economical for the mass production of thin-walled tubes.

However, the cold forging of thin-walled tubes is highly complex and influenced by various factors, including the shape of the tubes, the precision of the forging machine, the structure of the dies, and the skill level of the workers [10,11]. As a result of these in-fluences, various quality issues may arise in the production of the tubes, limiting their practical applications. For instance, cold forging of thin-walled circular tubes may result in oval-shaped cross-sections and uneven wall thickness. For thin-walled irregular tubes with non-circular cross-sections, the complexity of the required die structure increases, along with the higher performance requirements on the forging machine. Achieving high dimensional accuracy for these tubes becomes even more challenging [12,13,14]. Additionally, cold forging of thin-walled tubes may experience forming defects such as folding, cracks, and underfilling defects. Therefore, special attention should be paid to the tube forming quality.

To reduce forming damage and defects, the multi-stage cold forging technology has been widely applied when producing various products. This technology distributes the forging load through several pre-forming operations, effectively reducing its value and workpiece damage [15,16]. Lin et al. studied the four-stage cold forging of flange bolts and proposed the forging process [17]. To assess the damage to dies and bolt feasibility, the effective strain and forging force in each stage were investigated by finite element (FE) simulation. Finally, the authors confirmed the process feasibility. Jo et al. developed a multi-stage cold forging process for a one-body input shaft [18]. The FE simulation has shown that the produced shaft had no defects or fractures. Furthermore, experimental tests have shown that the mechanical properties of the shaft were increased by 45–50% compared to the machining process. Chen et al. used a multi-stage cold forging process to replace traditional machining process to produce a shell part [19]. Through FE analysis, effective strains and flow lines of the workpiece were investigated in different stages. Experimental work has shown that the geometrical quality and mechanical properties satisfied the technical requirements. Lee et al. proposed a six-stage cold forging process as an alternative to solenoid valve machining process [20]. Compared to machining, forging the billet reduced its weight by 46%.

The above-presented studies illustrate that an appropriate forging process is critical for obtaining high-quality products. For this reason, it is necessary to analyze the influence of the forging processes on the forming quality. However, the literature covering the multi-stage cold forging process of thin-walled tubes is scarce, with only a few available studies on rotary forging, hydroforging, and other similar tube forging processes.

Chen et al. introduced a double-roll pendulum hot rotary forging process for producing a large-diameter thin-walled ring [21]. The FE analysis was carried out to study the flow behavior, stress, strain, and temperature distribution of the ring. Experimental results have shown that the proposed process was feasible. Xu et al. employed a hydroforging process for forming thin-walled hollow parts with large deformation [22]. Thinning was observed at the free bulging stage, with wrinkles occurring in subsequent stages. It was concluded that selecting the appropriate internal pressure and loading path was essential. Simonetto et al. used hot rotary draw bending to manufacture thin-walled titanium alloy tubes [23]. Various heating strategies were investigated through the FE simulation, finding that selective heating was critical to the tube quality.

In this paper, we aimed to determine an appropriate multi-stage cold forging process of thin-walled A286 superalloy tube with ideal forming quality. Two forging processes were proposed based on the geometrical characteristics of the tube. The effects of the two forging processes on the tube forming quality were compared and analyzed via FE simulation. An appropriate forging process was selected based on the quality requirements. Optical microscope (OM), micro-hardness, scanning electron microscope (SEM) and electron backscattered diffraction tests (EBSD) were carried out to validate the forming quality of the tube forged using the selected process.

## 2. Materials and Methods

### 2.1. Forging Quality Requirement

**Figure 1:**
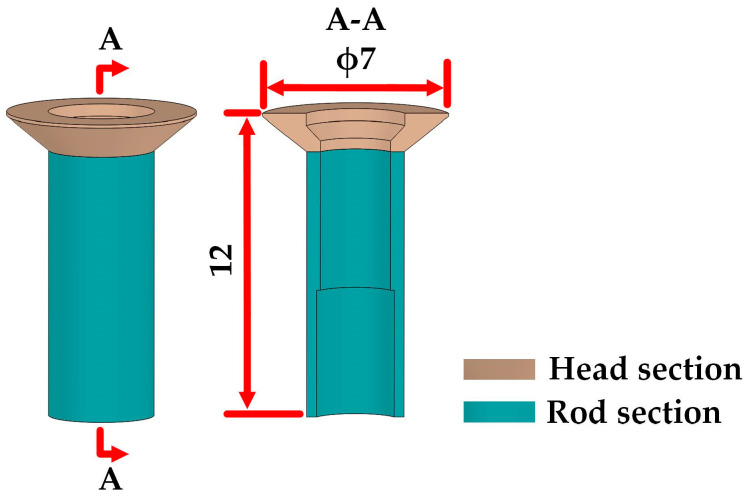
Schematic view of the thin-walled tube.

The thin-walled tube investigated in this study is an axisymmetric component characterized by a maximum outer diameter of 7 mm and a maximum length of 12 mm. It is composed of two sections: the head section located above, and the rod section located below. The geometry of the tube is depicted in Figure 1. Prior to use, the tube needs to be assembled in a pre-drilled hole. The outer taper of the head section serves a positioning function, while the rod section undergoes large plastic deformations to achieve the connection function. When working, the head section is fixed and acts as a load-bearing element. Therefore, the cold forged tube must meet the following quality requirements in order to ensure the load-bearing capacity and connection reliability: (1) no underfilling defects or cracks should be present in the head section; (2) the hardness of the head section should be greater than that of the rod section, and both sections should have hardness values exceeding 300 HV.

### 2.2. Multi-Stage Cold Forging of the Thin-Walled Tube

#### 2.2.1. Materials and Cold Forging Machine

Multi-stage cold forging technology was employed to manufacture the thin-walled tube for reducing forging load and damage. The initial billet is in the form of a cylindrical shape, featuring a diameter of 3.94 mm and a length of 6.7 mm. It was machined from a solution heat-treated A-286 superalloy with a hardness of 240 HV. This material was supplied in compliance with the AMS5731 standard, which specifies a heating temperature of 980 °C and a 2 h hold time for the solution treatment process [24]. During this treatment, the various phases within the material were completely dissolved, resulting in improved toughness and plasticity to meet the specific demands of cold forging operations. Table 1 lists the chemical composition of the A-286 superalloy.

W6Mo5Cr4V2 steel, known for its high hardness, excellent wear resistance, and impact resistance, was chosen as the die material [25,26]. The clearance between the workpiece and the bottom die was 0.02 mm.

A high-speed multi-stage cold forging machine with an effective load capacity of 392 KN was used to manufacture the thin-walled tube at room temperature. The machine is capable of processing 120–200 pieces of products per minute. During the forging process, mineral oil (China Petrochemical Corporation M0618, Beijing, China) was utilized as a lubricant to mitigate friction between the workpiece and the dies. The workpieces were transported using mechanical arm clamps, and ejected from the dies utilizing hydraulic ejector pins.

#### 2.2.2. Cold Forging Processes

**Figure 2:**
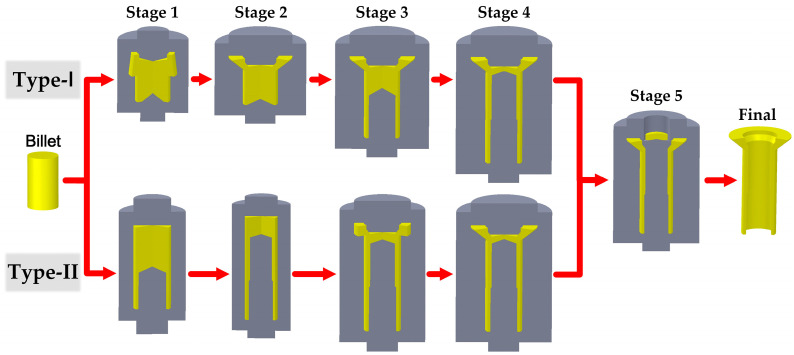
Schematic diagram of the proposed cold forging processes.

Based on the geometrical features of the thin-walled tube, we designed two types of multi-stage cold forging processes, designated as Type-I and Type-II. Both processes include five forging stages. The Type-I process consists of the following forging stages: head preforming, final head forming, first backward extrusion, second backward extrusion and piercing, while the forging stages of the Type-II process include first backward extrusion, second backward extrusion, head preforming, final head forming and piercing. The schematic diagram of the proposed forging processes is illustrated in Figure 2. Table 2 lists the forging stroke of the prime die during each forging stage.

In the Type-I process, the head section was forged first, followed by the rod section. In the first stage, a cylindrical billet was pre-formed to obtain a tapered head. Next, the tapered head was formed again to achieve a prescribed dimensional shape in the second stage. Then, the first backward extrusion was performed to create a blind hole at the workpiece bottom in the third stage. A smaller hole at the top of the blind hole was backward extruded in the fourth stage. Finally, a piercing operation was carried out to produce a through-hole.

The Type-II process features a reverse forging sequence compared to the Type-I process. In the first stage, the cylindrical billet was backward extruded to form a blind hole at the bottom of the workpiece. A second backward extrusion was conducted to produce a smaller hole at the top of the blind hole in the second stage. Next, a pre-forming operation was carried out to establish the basic shape of the head section in the third stage, followed by a final head-forming operation to create a prescribed dimensional head in the fourth stage. The fifth stage was identical to that of the Type-I process.

Predicting the effects of the forging processes on geometrical defects and hardness distribution of the workpiece is essential for obtaining a thin-walled tube with ideal quality. However, prediction is not feasible based solely on the experience of the process designers. Therefore, FE simulations were performed to gain a principal understanding of the proposed processes.

### 2.3. FE Modeling

#### 2.3.1. Material Properties and Constitutive Model

The proposed forging processes were simulated using DEFORM 3D v11.0 software. This software is specifically developed for simulating a diverse array of forging processes used in metal forming and related industries [27,28]. The diameter and height of the cylindrical billet were 3.94 mm and 6.7 mm, respectively. The A-286 superalloy was selected as the material of the billet. Its mechanical properties, obtained from the built-in software database, were adopted for the simulation. The W6Mo5Cr4V2 steel was chosen as the die material. Table 3 lists its mechanical properties [26]. In order to streamline the computational workload, the influence of material hardness and temperature on mechanical properties was not considered in the present study.

**Figure 3:**
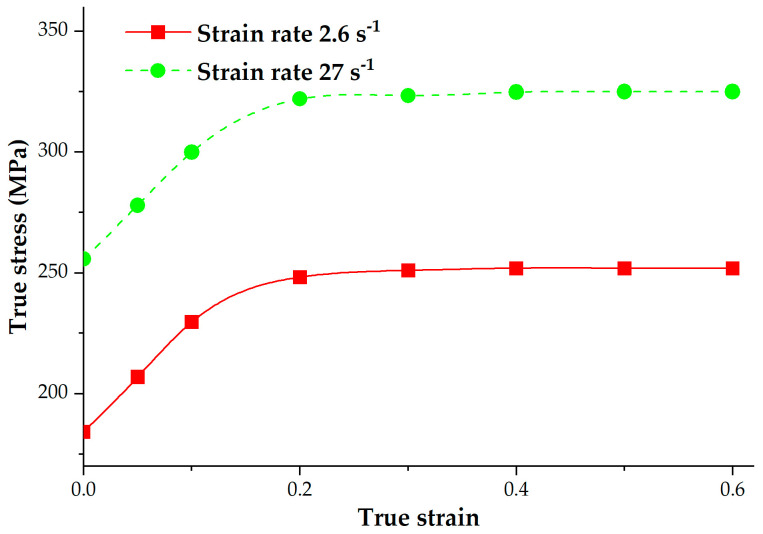
True stress–strain curves of the A-286 superalloy.

Figure 3 depicts the true stress–strain data of the A-286 superalloy. A Power Law model, which neglects the temperature influence, was employed to characterize the material’s flow stress. The Power Law model can be denoted by Equation (1),

(1) $$\bar{\sigma }=c{\bar{\varepsilon }}^{n}{\dot{\bar{\varepsilon }}}^{m}+y,$$

where $\bar{\sigma }$ is the flow stress, c denotes the material constant, m presents strain rate exponent, n is the strain exponent, y denotes the initial yield value, $\bar{\varepsilon }$ presents the effective plastic strain, $\dot{\bar{\varepsilon }}$ is effective strain rate. Table 4 lists the constants of the Power Law model.

#### 2.3.2. FE Model

**Figure 4:**
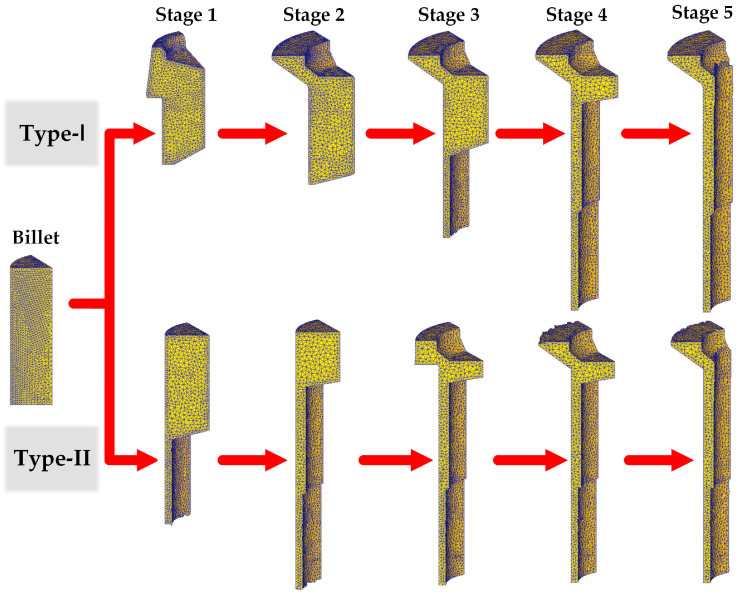
One-sixth FE models of the workpiece in different forging stages.

The solver type employed in the simulation was Lagrangian Incremental, which is highly suitable for conventional forming and extrusion applications [27,29]. Three-dimensional one-sixth FE models of the billet and dies were developed to improve simulation efficiency. In the model, the billet was set as a plastic body. Figure 4 presents the FE models of the workpiece in different forging stages. 3D Tetrahedral elements were employed to mesh the billet, yielding a total of 30,000 elements. An element size ratio of 2 was applied to refine the mesh in the large deformation region of the billet, ensuring a minimum element size of 0.078 mm and a maximum size of 0.156 mm. Autoremesh was activated to handle the remeshing of objects undergoing large plastic deformation. Volume compensation was utilized to maintain the volume of the deforming object. The dies were set as rigid bodies to avoid fracture failure under the high load conditions.

Penalty method and a constant shear factor of 0.08 were adopted to define the contact behavior between the dies and the workpiece [30]. The bottom die and ejector pin were fixed during the simulation. The prime die moved downward to forge the workpiece at a constant speed of 75 mm/s in each forming stage. Displacement step increment was defined as 0.1 mm/step to carefully analyze the changes in the workpiece’s mechanical performance. The forming stroke of the prime die used in simulation was set according to the values provided in Table 2.

### 2.4. Test and Characterization

The billet and the forged thin-walled tube used for tests were initially cut by wire electrical discharge machining (WEDM, Minkes DK7745, Taizhou, China) into longitudinal cross-sectional samples. Next, the cross-sectional tube was further divided into the rod section and head section (illustrated in Figure 1) using WEDM.

**Figure 5:**
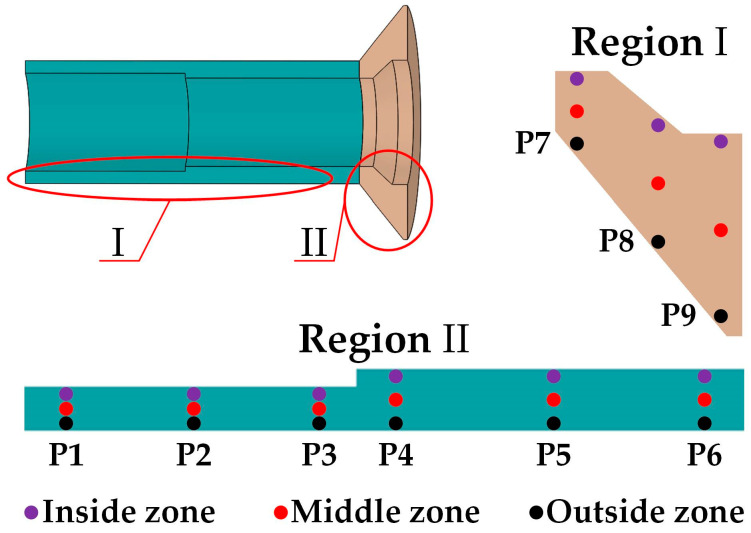
Schematic of locations for hardness measurement and effective strain extraction.

The hardness distribution of the final forged thin-walled tube was measured using a Vickers hardness tester (Huarui HVZ-1000ZQ, Jinan, China) with a 0.1 kg load and a 15 s dwell time. The samples for hardness measurement were mechanically grounded and polished. Figure 5 illustrates a schematic of the locations for hardness measurement. The hardness distribution was tested at the inside, middle, and outside zones of the nine positions (P1 to P9) of the tube. A total of 27 measurement dots were employed for hardness testing. Each dot was measured three times, and the average value was used for hardness analysis to minimize testing errors. Moreover, the dots in Figure 5 also indicate the locations for extracting effective strain values from simulation results.

A scanning electron microscope (SEM, ZEISS Sigma 300, Oberkochen, Germany) was utilized to examine the micro-crack within the head section of the experimental thin-walled tube, with an accelerating voltage of 3 kV. SEM images were obtained at the midpoint and stress concentration points within the head section. The specimens underwent mechanical grinding and polishing prior to SEM observation.

Kernal Average Misorientation (KAM) maps and microstructure of the experimental thin-walled tube were measured using electron backscatter diffraction detector (EBSD, EDAX Hikari Plus, Pleasanton, CA, USA) with an accelerating voltage of 30 kV and a scanning step size of 0.25 μm. TSL OIM Analysis 6.2 software was used to process the collected EBSD data. The dots in the inside zone of the two positions (P2 and P8) shown in Figure 5 and the dot in the center position of the billet were selected for EBSD analysis. The specimens for EBSD observation were initially subjected to mechanical grinding and polishing with the same method as previously described. Next, the polished specimens were electrolytically corroded in a mixture of 180 mL of C_2_H_5_OH + 20 mL of HClO_₄_ for a duration of 30 s at a stable voltage of 28 V. Finally, the specimens were washed using ethanol.

The flow line distribution of the forged tube was investigated using optical microscopy (OM, Sunny ICX41M, Ningbo, China). Three optical images of the tube were captured to observe the flow line distribution. The specimens utilized for OM underwent initial mechanical grinding and polishing, followed by chemical etching with a reagent containing 30 mL of HCl and 10 mL of HNO_3_.

## 3. Results

### 3.1. Influence of the Forging Processes on Forming Quality

#### 3.1.1. Forging Force

To assess the forgeability of the workpieces manufactured using the two processes, we compared the forging forces in each forming stage. Given that the forging conditions in Stage 5 during the two processes are identical, our analysis was specifically directed towards the forces in the initial four stages.

**Figure 6:**
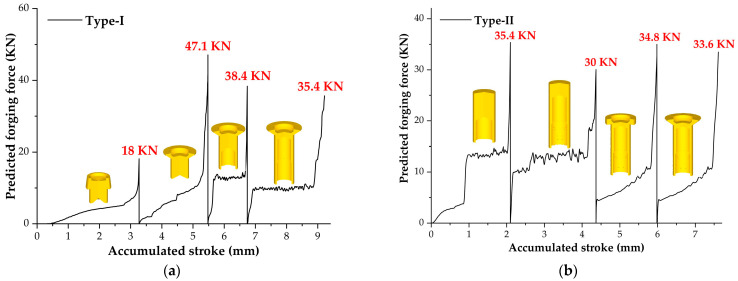
Predicted forging force of the prime die: (**a**) Type-I process; (**b**) Type-II process.

Figure 6 shows the predicted forging force for the two processes derived from the simulation results. In general, the force variations exhibited similar trends in the different forging stages. (1) In the initial phase of the forging stage, a substantial increase in force was observed. The reason for this phenomenon is that the dies and the workpiece surface were just in contact. As the prime die moved, the contact area between the dies and the workpiece rapidly expanded, leading to a significant amplification in the degree of workpiece deformation. (2) In the middle phase of the forging stage, the force growth rate decelerated, potentially due to the stabilization of the contact area between the dies and the workpiece. (3) In the final phase of the forging stage, there was a sharp surge in the force. The gap between the workpiece and the dies rapidly decreased in this phase, while the die cavity remained closed. Consequently, the resistance to deformation during material flowed increased sharply.

The maximum forces in each forging stage during the Type-I process were 18 KN, 47.1 KN, 38.4 KN, and 35.4 KN, respectively. In comparison, the corresponding values for the Type-II process were 35.4 KN, 30 KN, 34.8 KN, and 33.6 KN, respectively. It is evident that the Type-II process exhibited a lower maximum load compared to the Type-I process. In addition, the maximum loads in each forging stage were relatively close to each other for the Type-II process. This characteristic provides an advantage in terms of improving the service life of the dies.

#### 3.1.2. Geometrical Defect

**Figure 7:**
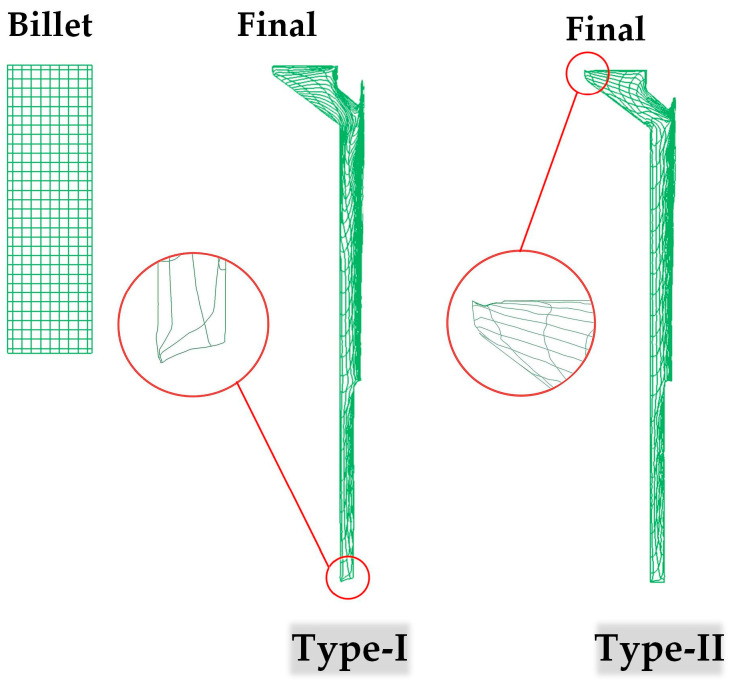
Simulated flow lines of the forged thin-walled tube prepared by the proposed processes.

Figure 7 illustrates the simulated flow lines of the final thin-walled tube forged using the proposed processes. Both processes exhibited undistorted flow lines in general. Dense flow lines were observed on the inner surface due to the friction and shear effects caused by the punch and pin. Furthermore, underfilling defects were identified in the tube as well. The Type-I process exhibited an underfilling defect at the bottom side of the rod section, whereas for the Type-II process, an underfilling defect was found at the top side of the head section. This discrepancy in defect location can be attributed to the reverse flow direction of the material caused by different forging sequences. Here are the specific reasons: (1) For the Type-I process, the final shaping of the rod section occurred in Stage 4. The material in the rod section flowed downward along the axial direction. Consequently, the axial gap between the bottom die and the bottom end of the rod section was filled in the final phase, which likely led to underfilling defects in this specific area. (2) For the Type-II process, the material in the head section began to flow diagonally upward to fill the gap between the top of the head section and the top die cavity in the final phase of Stage 4. This flow behavior can be considered as the primary reason for the observed defect. Since the dimensional accuracy of the product is negatively affected by the presence of defects, the head section in the tube prepared by the Type-I process may have higher geometrical quality.

**Figure 8:**
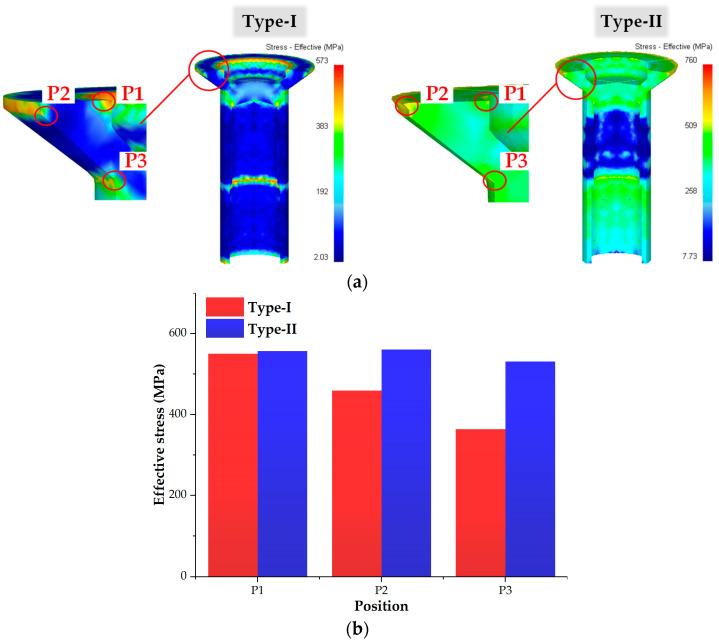
Effective stress of the workpiece in Stage 4 in the proposed processes: (**a**) effective stress distribution; (**b**) stress values at different positions.

It is well known that areas of local stress concentration are prone to crack initiation [31,32]. As shown in Figure 8a, the stress concentration in the head section of the tube prepared by the Type-I process was lower than the tube prepared by the Type-II process. For both processes, the edge of the hole (P1), the upper edge of the outer cone (P2) and the transition zone between the cone and the rod section (P3) showed evident stress concentrations.

To quantitatively evaluate the stress concentration, stress values at three positions (P1 to P3) were extracted and are presented in Figure 8b. For the Type-I process, the stress values at P1, P2 and P3 were 550 MPa, 459 MPa and 364 MPa, respectively. The corresponding values for the Type-II process were 557 MPa, 561 MPa and 531 MPa, respectively. Compared to the Type-II process, the stress for the Type-I process was slightly lower at P1, and significantly lower at P2 and P3. Two factors were responsible for the occurrence of this phenomenon. (1) During Stage 4 of the Type-I process, the material in the rod section underwent axial downward flow, effectively alleviating the stress concentration in the head section. (2) For the Type-I process, the forging force was primarily applied to shape the rod section in Stage 4, while the corresponding force for the Type-II process was mainly utilized to form the head section. It can be inferred that the material in the head section for the Type-I process experienced relatively lower forces compared to the Type-II process. Therefore, the Type-I process can minimize the stress concentration in the head section, which may lead to a lower probability of crack initiation.

#### 3.1.3. Effective Strain and Hardness Distribution

**Figure 9:**
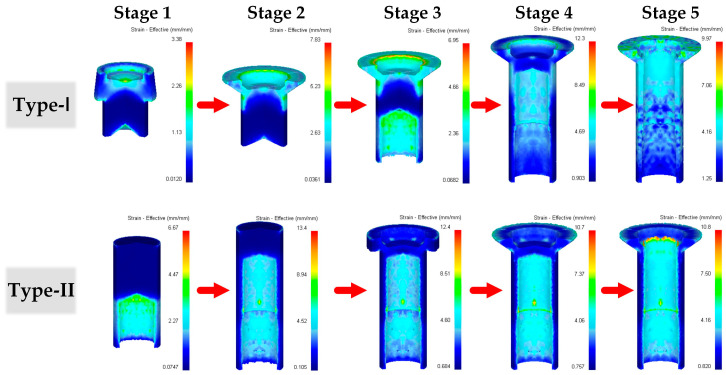
Effective strain distribution in the workpiece during different forging stages.

The amount of plastic strain directly affects hardness distribution in the forged product [33,34]. Higher effective strains yield larger hardness values [35]. Consequently, the influence of the two forging processes on the effective strain distribution was investigated to predict the difference in hardness of the final forged thin-walled tube. Figure 9 shows the effective strain distribution in the workpiece in different forging stages during the proposed processes. Non-uniform distribution of effective strain throughout the workpiece was observed in all five stages. For the Type-I process, high effective strain regions were mainly concentrated in the head section. In contrast, regions with relatively low effective strain were observed in the head section for the Type-II process. The observed phenomenon can be attributed to the varying deformation levels of the workpiece, which were caused by different forging sequences. Specifically in the Type-I process, the head section underwent four forging operations (from Stage 1 to Stage 4), whereas in the Type-II process, it was forged only twice (Stage 3 and Stage 4). The greater number of forging operations in the Type-I process resulted in a higher level of material deformation in the head section compared to the Type-II process.

**Figure 10:**
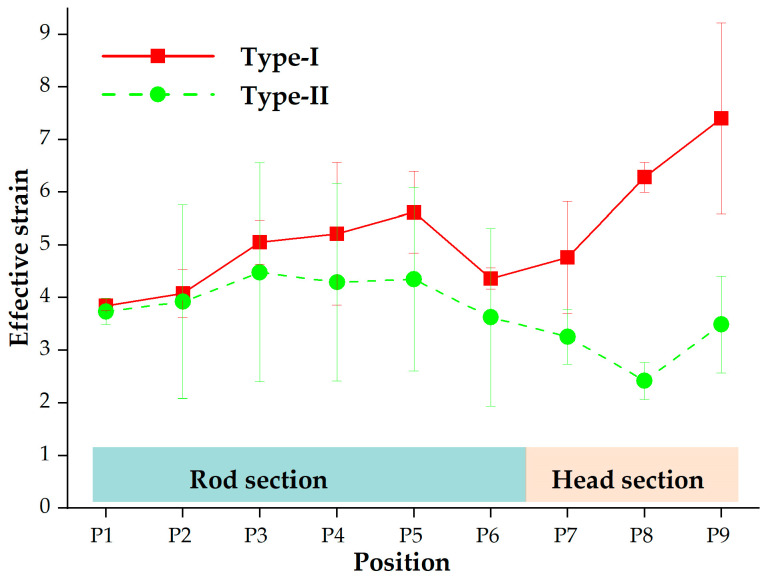
Effective strain values of the forged thin-walled tube using the proposed processes.

The effective strain values of the final forged tube were extracted to quantitatively estimate the difference in the effective strain distribution between the two processes. The positions for strain extraction, depicted in Figure 5, are the same as those used for hardness measurement. Figure 10 shows the extracted effective strain values. The effective strains for the Type-I process were overall larger than those for the Type-II process. Furthermore, the effective strain in the head section for the Type-I process varied in the range of 4.11 to 8.47, while the corresponding strain magnitudes in the rod section ranged from 3.65 to 6.77. The effective strain in the head section was generally greater than that in the rod section. Due to the relationship between effective strain and hardness, the head section prepared by the Type-I process may have higher hardness magnitudes compared to the rod section.

### 3.2. Selection of the Appropriate Forging Process

Based on the simulation results discussed in Section 3.1, the head section of the final forged thin-walled tube prepared by the Type-I process was defect-free and exhibited higher effective strain magnitudes. This may result in the head section having superior geometrical quality and higher hardness magnitudes when compared to the rod section. Although the forging force required for the Type-I process was higher than that for the Type-II process, leading to a reduction in die life, it remained significantly lower than the load capacity of the cold forging machine. This indicates that the selected forging machine is capable of producing the tube using the Type-I process. Therefore, the Type-I process was selected as the appropriate forging process to meet forming quality requirements.

### 3.3. Experimental Validation

#### 3.3.1. Geometrical Quality

**Figure 11:**
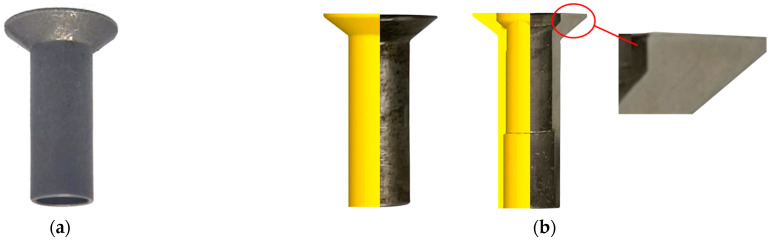
Configuration of the experimental and simulated thin-walled tube prepared using the Type-I process: (**a**) final experimental thin-walled tube; (**b**) comparison of configuration between the simulated and experimental tube.

An experimental thin-walled tube was successfully forged using the Type-I process, as shown in Figure 11a. Given that the underfilling defects specified in the quality requirement are macroscopic in nature, we conducted a direct visual inspection of the exterior of the experimental tube. No apparent underfilling defect was observed in this tube. In order to further confirm the geometrical quality of the tube, we analyzed the cross-sectional configuration of the head section. Figure 11b compares the simulated and experimental configurations of the thin-walled tube. The configurations of the experimental tube were in good agreement with their simulated counterparts. No underfilling defect was found in the cross-section of the head section, confirming the defect prediction by simulated flow lines.

**Figure 12:**
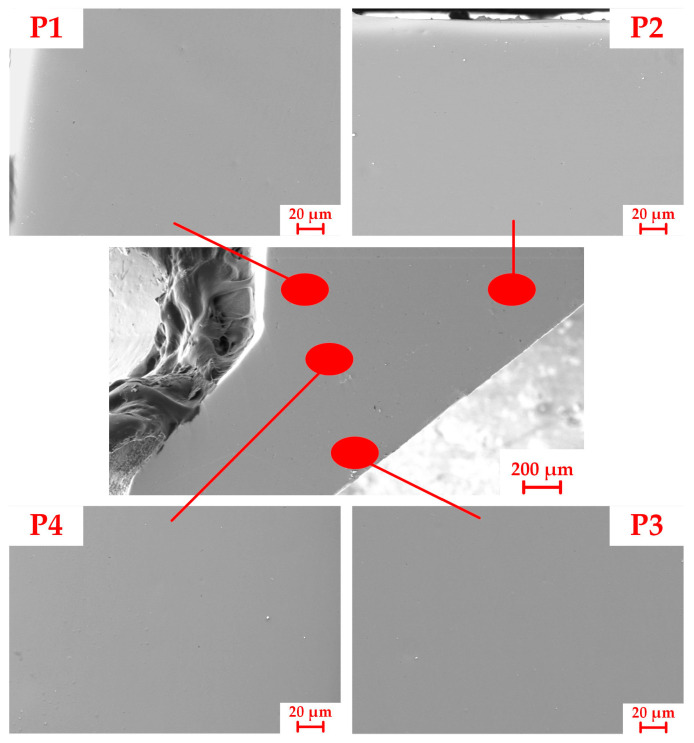
SEM images collected from the head section.

SEM was used to thoroughly examine the entire head section of the experimental tube for any microscopic defects. Apart from the presence of some abrasive particles, no significant microcracks were detected. Thus, the final product satisfies the geometrical quality requirement on the head section. Due to limitations in space, only five representative SEM images at various magnifications were captured and shown in Figure 12. These images served as a representation of the observed findings. P1–P3 corresponded to stress concentration positions identified in the simulation and P4 represented the central position of the head region.

#### 3.3.2. Hardness Distribution

**Figure 13:**
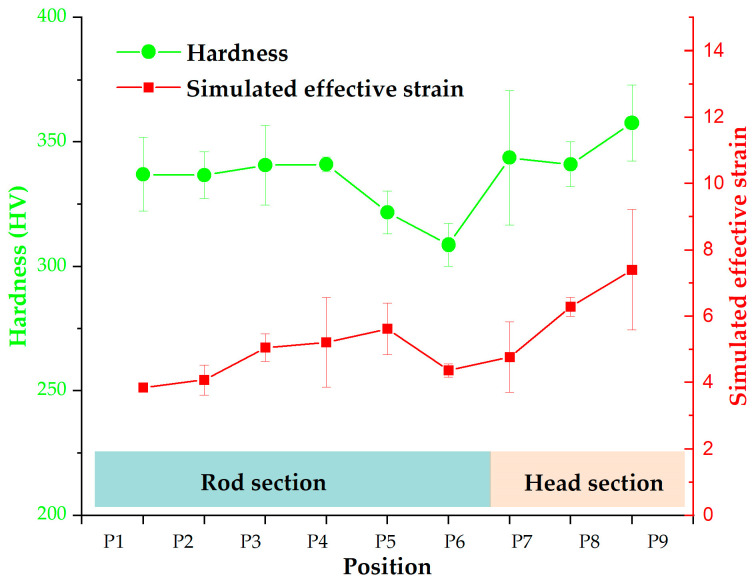
Experimental hardness and simulated effective strain values of the final thin-walled tube forged using the Type-I process.

The hardness distribution of the experimental thin-walled tube forged using the Type-I process was measured at locations outlined in Figure 5. The hardness value at each position was calculated by averaging the values for the inside, middle, and outside zones. Figure 13 displays the average, maximum, and minimum hardness values at different positions of the experimental tube. The hardness range in the rod section ranged from 300 to 354 HV, followed by an increase with a range of 314 to 371 HV in the head section. Compared with the billet (240 HV), the hardness value in the head section increased by up to 55%. The high hardness of the head section ensured the high reliability of the tube. Thus, the tube meets the quality requirements for hardness.

The experimental hardness distribution generally exhibited similarities to the simulated strain distribution, showing a lower trend in the rod section and a higher trend in the head section. Hence, comparing the simulated strain distributions in thin-walled tubes is a viable approach to predict the variations in hardness distribution for different forging processes.

#### 3.3.3. Flow Lines

**Figure 14:**
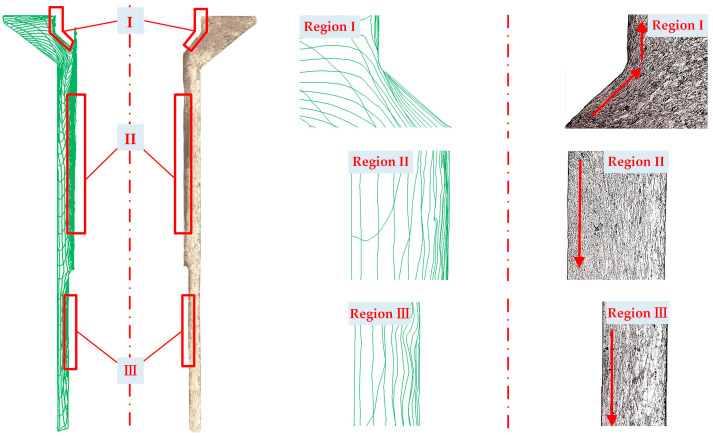
Comparison of the simulated and experimental flow lines of the final thin-walled tube forged using the Type-I process.

Figure 14 shows a comparison between the flow lines obtained experimentally and through simulations. The experimental flow lines were found to be consistent with the simulation results. Denser flow lines were observed on the inner surface of the three regions from the simulation and experimental results. Furthermore, the flow lines first moved upward at an angle of 35°, then followed along the longitudinal direction in the head section (Region I). The flow direction was observed to be downward in the rod section (Regions II and III).

#### 3.3.4. Strain Distribution

**Figure 15:**
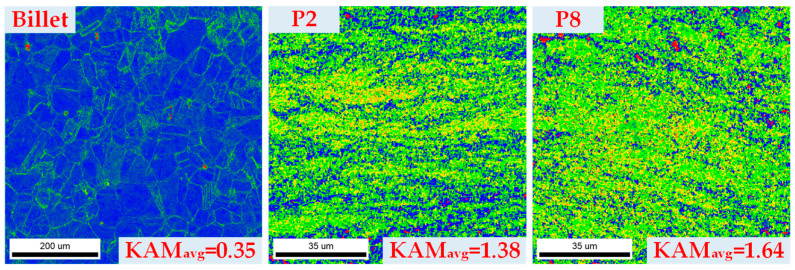
EBSD analysis of KAM maps of the billet and experimental thin-walled tube forged using the Type-I process.

The Kernal Average Misorientation (KAM) map was used to estimate the local strain concentration after forging, where a high KAM value corresponds to a high strain [36,37]. Figure 15 shows the KAM distribution for the billet and the thin-walled tube forged using the Type-I process. The average KAM values (KAM_avg_) for the billet, rod section (P2), and head section (P8) were 0.35, 1.38 and 1.64, respectively. Compared with the billet, the KAM_avg_ value of the rod section and the head section increased by 294% and 368%, respectively. The KAM_avg_ value for the head section was higher than that of the rod section. This indicates that the head section has a higher local strain level compared to the rod section, which agrees well with the simulation results.

#### 3.3.5. Microstructure Evolution

**Figure 16:**
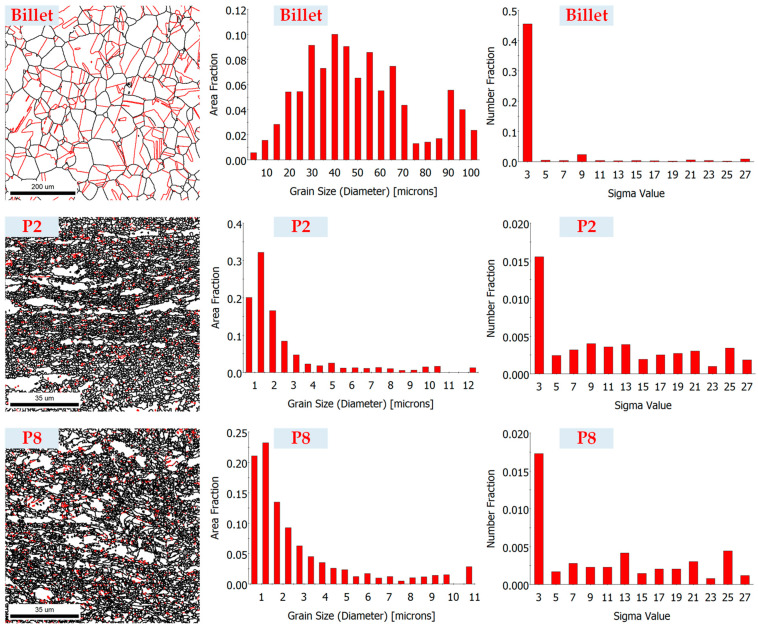
EBSD analysis of microstructure of the billet and experimental thin-walled tube forged using the Type-I process.

Figure 16 illustrates the microstructure of the billet and the thin-walled tube forged using the Type-I process. The billet exhibited uniform equiaxed grains, with an average diameter of 21.38 μm. After cold forging, the microstructure underwent significant refinement. The head section (P8) showed an average grain diameter of 0.85 μm, which was smaller than that of 1.05 μm for the rod section (P2). Consequently, the Type-I process effectively refined the grain of the material. However, the discrepancies in grain size distribution indicate that the material experienced non-uniform deformation, with a higher level of plastic deformation and strain in the head section (P8) compared to the rod section (P2).

In Figure 16, it can also be observed that a significant proportion of ∑3 annealing twins (indicated by the red line) was present within the microstructure of the as-solutioned billet, reaching a fraction of 45.5%. After the billet was forged, there was a substantial reduction in the presence of ∑3 annealing twins. The proportion of ∑3 annealing twins in the rod section and head section (P8) decreased to 1.5% and 1.7%, respectively.

## 4. Conclusions

Two multi-stage cold forging processes for manufacturing a thin-walled tube were proposed in this work. The effects of the processes on the forming quality of the forged tube were analyzed using FE simulations and experimental studies. The following conclusions can be drawn:

(1) The Type-I process was selected as the suitable forging process as it met the quality requirements on the thin-walled tube based on the simulation results. The Type-I process consists of the following forging stages: head preforming, final head forming, first backward extrusion, second backward extrusion, and piercing.

(2) The Type-II process exhibited two primary issues. Firstly, there was a noticeable underfilling defect observed at the top side of the head section, which did not meet the required geometric quality requirement. Secondly, the strain distribution in the head section was lower compared to that in the rod section, potentially leading to a reduced hardness distribution in the head section.

(3) An experimental thin-walled tube meeting the quality requirement was successfully forged using the Type-I process, confirming the feasibility of the selected process. No noticeable underfilling defects or microcracks were observed in the head section. The hardness distribution in the head section generally exceeded that of the rod section. The simulation results agreed well with the experimental results.

(4) That the Type-I process imposed more severe plastic deformation on the material in the head section may be the main reason for the increased hardness in this section. The head section exhibited a higher level of grain refinement compared to the rod section. Additionally, a significant amount of ∑3 annealing twins within the material reduced after cold forging.

## Figures and Tables

**Table 1:** Chemical composition of the A-286 superalloy (wt.%).

Ni	Cr	Mo	Mn	Co	C	B	Ti	Al	V	Cu	Si	Fe
24.7	15.2	1.3	1.5	0.19	0.05	0.001	2.1	0.29	0.28	0.03	0.6	Bal

**Table 2:** Forging stroke of the prime die during each forging stage.

Forging Stroke/mm					
Forging Process	Stage 1	Stage2	Stage 3	Stage 4	Stage 5
Type-I	3.27	2.21	1.26	2.49	2.67
Type-II	2.09	2.27	1.62	1.63	2.67

**Table 3:** Mechanical properties of the billet and dies.

Parameters	Unit	Billet	Dies
Material	-	A-286	W6Mo5Cr4V2
Density	g/cm^3^	7.92	7.81
Young’s modulus	MPa	206,754	212,000
Poisson’s ratio	-	0.3	0.29

**Table 4:** Constants of the Power Law model.

Constant	c	m	n	y
Value	9.99802	0.732542	0.231611	234.407

## Data Availability

Not applicable.
